# Assisted Reproductive Technology Treatment, the Catalyst to Amplify the Effect of Maternal Infertility on Preterm Birth

**DOI:** 10.3389/fendo.2022.791229

**Published:** 2022-06-02

**Authors:** Youzhen Zhang, Wei Zhou, Wanbing Feng, Jingmei Hu, Kuona Hu, Linlin Cui, Zi-Jiang Chen

**Affiliations:** ^1^Center for Reproductive Medicine, Cheeloo College of Medicine, Shandong University, Jinan, China; ^2^Key Laboratory of Reproductive Endocrinology of Ministry of Education, Shandong University, Jinan, China; ^3^Shandong Key Laboratory of Reproductive Medicine, Jinan, China; ^4^Shandong Provincial Clinical Research Center for Reproductive Health, Jinan, China; ^5^National Research Center for Assisted Reproductive Technology and Reproductive Genetics, Shandong University, Jinan, China; ^6^Shanghai Key Laboratory for Assisted Reproduction and Reproductive Genetics, Shanghai, China; ^7^Center for Reproductive Medicine, Ren Ji Hospital, School of Medicine, Shanghai Jiao Tong University, Shanghai, China

**Keywords:** assisted reproductive technology, infertility, ovulatory dysfunction, neonatal outcome, preterm birth

## Abstract

**Objective:**

To identify the influence of different infertility causes and assisted reproductive technology (ART) treatment on perinatal outcomes and clarify the relationship between the maternal pathophysiological changes and artificial interventions.

**Methods:**

A total of 1,629 fertile women and 27,112 infertile women with sole infertility causes were prospectively recruited from July 2014 to December 2017, and 9,894 singletons were finally enrolled into the study. Pregnancies with more than one cause of infertility and/or multiple births were excluded. According to the causes of infertility and the exposure of ART treatment, the participants were divided into four groups, namely, fertile naturally conceived (NC) group, infertile NC group, female factor ART group, and male factor ART group. Perinatal outcomes, including gestational age of delivery (GA), birth weight (BW), preterm birth (PTB), low birth weight (LBW), small for gestational age (SGA), and large for gestational age (LGA), were compared among groups. Logistic regression was performed for the adjustment of several covariates.

**Result(s):**

The birth outcomes of the infertile NC group and fertile NC group, female factor ART group, and infertile NC group were comparable. Compared to the fertile NC group, the female factor ART group had a shorter GA (39.0 ± 1.6 vs. 39.3 ± 1.5 weeks, BW: P < 0.05). An interaction test showed that ART treatment had an interaction on the effect of female infertility on GA (P = 0.023). The female factor ART group also had a higher risk of PTB (OR 1.56, 95% CI 1.18–2.07) and LGA (OR 1.27, 95% CI 1.10–1.47) compared to the fertile NC group. The risk of PTB was increased for tubal factor ART (OR 1.49, 95% CI 1.12–2.00), ovulatory dysfunction ART (OR 1.87, 95% CI 1.29–2.72), and unexplained infertility ART (OR 1.88, 95% CI 1.11–3.17). The risk of LGA was increased for tubal factor ART (OR 1.28, 95% CI 1.11–1.48) and ovulatory dysfunction ART (OR 1.27, 95% CI 1.03–1.57).

**Conclusion(s):**

Our findings indicated that ART treatment could amplify the adverse effect of female infertility on neonates. Women with tubal factor infertility, ovulatory dysfunction, and unexplained infertility have a higher risk of PTB after ART treatment. Thus, clinicians should be vigilant in such patients and provide corresponding prevention strategies before and during pregnancy.

## Introduction

Infertility, defined as a lack of clinical pregnancy after regular unprotected sexual intercourse for at least 12 months, affects nearly 50 million couples worldwide (1, 2). The main causes of infertility include tubal factor, ovulatory dysfunction, endometriosis, unexplained infertility, and male factor (3, 4). Assisted reproductive technology (ART) has developed rapidly in the past few decades, solving the problem of infertility for most couples. Previous studies have suggested an association between the use of the ART technique and the risk of adverse perinatal outcomes in singletons, including preterm birth (PTB), low birth weight (LBW), small for gestational age (SGA), and fetal growth restriction (FGR), compared to pregnancies obtained through spontaneous conceptions (5). However, such association has not been fully confirmed in twin pregnancies, since many inconsistent findings have emerged from studies published in the last two decades (6–8). It may be that the increase in the risk of maternal and perinatal complications in multiple pregnancy hides any possible effect of the mode of conception on these outcomes (9).

The causes of deteriorated perinatal outcomes of ART offspring have not been well illustrated until now due to the confounding effect of the pathophysiological changes related to infertility. Previous studies have demonstrated that neonates of infertile women who conceived without ART treatment also showed an increased risk of PTB, LBW, and SGA compared with naturally conceived (NC) fertile women (10–12). This suggests that infertility may also contribute to the increased risk of adverse perinatal outcomes. Yet, the significant heterogeneity of such population indicates possible various influences of different infertility causes. Limited published studies have reported an association of increased PTB and SGA risks with maternal tubal disorders and ovulatory dysfunction (12, 13). Yet, the conclusion is still controversial with opposite findings (12, 14, 15). Furthermore, as additional exposure, whether the ART treatment aggravates the above association is unknown.

The aim of the present study is to identify the influence of different infertility causes and ART treatment on perinatal outcomes and clarify the relationship between the maternal pathophysiological changes and artificial interventions based on a prospective cohort. The results would be valuable for recognizing the ART patients with a high risk of poor perinatal outcomes and giving suggestions for prevention before or during pregnancy.

## Materials and Methods

### Participants

We conducted a prospective cohort study at the Hospital for Reproductive Medicine Affiliated to Shandong University. Recruitment started in July 2014 and ended in December 2017. Women who had registered in our reproductive center and planned a pregnancy were recruited into our research. During the same period, fertile women were recruited from the obstetrics clinic in the first trimester. Eligible women were over 18 years old, residents of China, fertile or infertile with single infertility-related diagnosis, and willing to accept the follow-up visits. They were phone interviewed by a group of trained nurses at the end of the first trimester and after puerperium. Baseline information was recorded before pregnancy or at the end of the first trimester. At the end of the first trimester, the participants were asked if they were clinically pregnant, ensuring whether they could continue being followed. Their complications during pregnancy, delivery medical records, and hospital discharges were obtained after the puerperium. During the follow-up, the participants were removed from the cohort according to the following exclusion criteria: 1) without a live birth, such as no clinical pregnancy, abortion, or stillbirth; 2) multiple births; 3) lack of neonatal information; 4) gestational age at delivery is less than 22 weeks or birth weight is less than 500 g.

### Exposure Assessment

The exposure factor was female infertility or ART treatment, both of which were obtained from the participants’ medical records at the end of the first trimester. Therefore, there were three exposed groups consisting of infertile NC women, female factor ART-treated women, and male factor ART-treated women. Fertile NC women were set as the unexposed group. According to the causes of infertility, the infertile ART women were further divided into five subgroups, namely, tubal factor ART group, ovulatory dysfunction ART group, endometriosis ART group, unexplained infertility ART group, and male factor ART group. The cause of infertility was diagnosed by clinical doctors in our center. Tubal factor was diagnosed through hysterosalpingography or laparoscopy, including peritubal adhesions, tubal obstruction, and hydrosalpinx. Ovulatory dysfunction was defined as an irregular menstrual cycle or clinical evidence of oligoovulation or anovulation (16). Endometriosis was diagnosed through laparoscopy or ultrasound. Unexplained infertility was diagnosed based on normal results of semen analyses, assessments of ovulation, and hysterosalpingogram, according to the Practice Committee of the American Society for Reproductive Medicine (17). Male factor was diagnosed by one or more abnormalities through semen analyses based on the WHO Laboratory Manual for the Examination and Processing of Human Semen, 5th edition (18).

### Covariate Assessment

Body mass index (BMI) was calculated by dividing weight by height squared. Age at delivery was obtained from delivery medical records through puerperium follow-up. Information on parity and history of prior PTB was collected through questionnaires during follow-up. Maternal complications during pregnancy included pregnancy-induced hypertension (PIH) and gestational diabetes mellitus (GDM). PIH was defined as a new hypertension which appears at 20 weeks or a longer gestational age of pregnancy with or without proteinuria. GDM was defined as any degree of glucose intolerance with onset or first recognition during pregnancy.

### Outcome Assessment

Gestational age at delivery (GA, week) was calculated according to the last menstrual period (LMP) and confirmed by fetal crown-rump length measurement at the first trimester through ultrasound in NC pregnancies (19). In ART pregnancies, it was accurately estimated based directly on the date of fertilization. Birth weight (BW, g) was calculated by the midwife *via* a calibrated baby scale within 1 h after delivery. Deliveries were classified as PTB if GA was <37 weeks. LBW was defined as birth weight <2,500 g regardless of GA. SGA and large for gestational age (LGA) were defined respectively as weight less than the 10th percentile or larger than the 90th percentile of according gestational age and sex. The BW z-score was calculated based on the Chinese standard for sex- and gestational age-specific birth weight (20).

### Statistical Analysis

All analyses were performed using SPSS (Statistical Packages for The Social Sciences) software version 24 (SPSS Inc., Chicago, IL, USA). The continuous variables were first evaluated for the normality of statistical distribution by graphically using the QQ plot. Descriptive statistics were expressed as mean ± standard deviation (SD) and number (percentage %). Differences among groups were analyzed through the analysis of variance (ANOVA) (mean, SD) for continuous variables. The differences between groups were compared by the Games–Howell test or Tukey *post hoc* test for continuous variables. The chi-squared or Fisher’s exact test was used to analyze the differences among groups and differences between groups for categorical variables (n, %). The interaction test between maternal infertility and ART treatment was performed through two-way ANOVA. The logistic regression was performed to adjust for maternal age, BMI, GDM, and PIH. P < 0.05 was considered statistically significant.

### Ethics Statement

The study was approved by the Reproductive Medicine Ethics Committee, Hospital for Reproductive Medicine Affiliated to Shandong University. All participants signed written informed consents. The ethics approval number was No. 2014 (15).

## Results

### Population Characteristics

**Figure 1:**
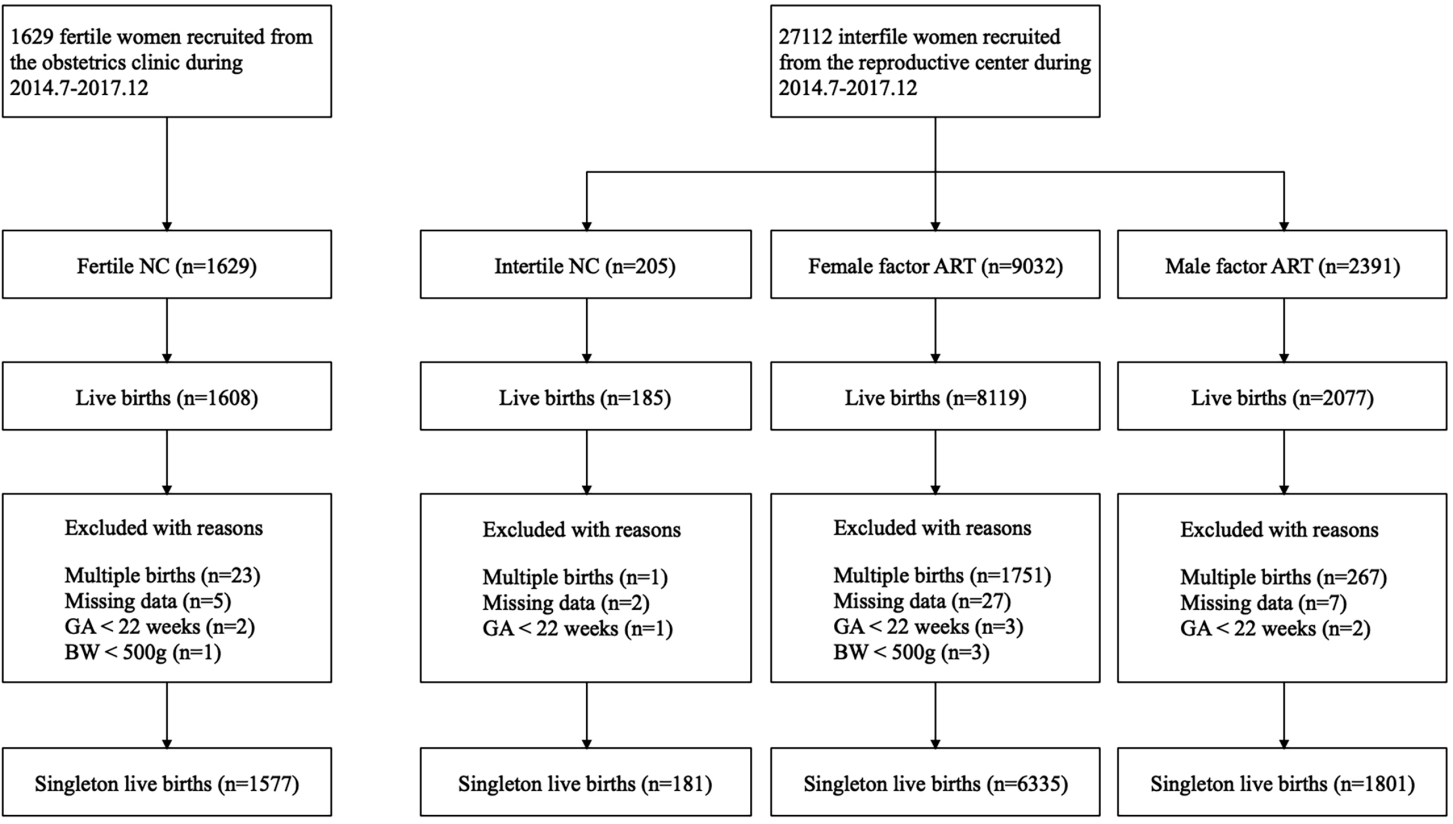
Participants in the cohort.

From July 2004 to December 2017, 1,629 fertile women and 27,112 infertile women were prospectively recruited. Up to March 2019, 11,989 of them had live births. We excluded 2,042 women with multiple births and 41 women with missing neonatal data. In accordance with the WHO recommendations (21), analyses were restricted to births whose GA were 22 weeks or longer, with a BW of at least 500 g, resulting in 12 women being excluded from the final analysis. The final study population was composed of 9,894 women who had singleton live births, including 1,577 fertile NC, 181 infertile NC, 6,335 female factor ART, and 1,801 male factor ART. The female factor ART group could be subdivided according to the causes of infertility, namely, tubal factor ART (n = 5,217), ovulatory dysfunction ART (n = 758), endometriosis ART (n = 87), and unexplained infertility ART (n = 273). A flowchart is presented in **Figure 1**. Demographic characteristics, including age, BMI, and pregnancy complications, were significantly different among the fertile NC group, infertile NC group, female factor ART group, and male factor ART group (**Table 1**). Although the proportion of women with parity of two or more was statistically different in each group (P = 0.021), there was no significant difference in the proportion of nulliparous or women with previous PTB history in each group (nulliparous P = 0.489; previous PTB P = 0.564). Compared to the fertile NC group, women in the infertile NC group had an older age at delivery and a lower BMI before pregnancy (age: 32.3 ± 3.6 vs. 28.9 ± 4.3 years, P < 0.05; BMI: 22.9 ± 3.2 vs. 23.6 ± 3.7 kg/m^2^, P < 0.05), while they showed a comparable incidence of GDM and PIH (GDM: 5.0% vs. 4.0%, P > 0.05, PIH: 1.7% vs. 2.9%, P > 0.05). Women in the female factor ART group were older than those in the fertile NC group and had a higher incidence of GDM and PIH (age: 31.7 ± 4.4 vs. 28.9 ± 4.3 years, P < 0.05; GDM: 6.3% vs. 4.0%, P < 0.05; PIH: 4.5% vs. 2.9%, P < 0.05).

**Table 1:** Baseline characteristics and birth outcomes.

Factor	Fertile NC	Infertile NC	Female factor ART	Male factor ART	P	P*^i^*
**N**	1,577	181	6,335	1,801		
**Female age (y), mean (SD)*^abce^***	28.9 ± 4.3	32.3 ± 3.6	31.7 ± 4.4	30.4 ± 4.5	<0.001*^f^*	–
**Female BMI (kg/m^2^), mean (SD)*^abc^***	23.6 ± 3.7	22.9 ± 3.2	23.0 ± 3.4	22.9 ± 3.5	<0.001*^f^*	–
**Male age (y), mean (SD)*^abcde^***	30.1 ± 4.7	33.2 ± 4.1	31.9 ± 4.7	31.4 ± 5.2	<0.001*^f^*	–
**Male BMI (kg/m^2^), mean (SD)*^abc^***	24.7 ± 3.6	25.5 ± 3.5	25.7 ± 3.9	25.8 ± 4.1	<0.001*^f^*	–
**Parity (n, %)**						
** ≥2*^c^***	17 (1.1%)	2 (1.1%)	44 (0.7%)	5 (0.3%)	0.021*^g^*	–
** 1**	252 (16.0%)	25 (13.8%)	967 (15.3%)	268 (14.9%)	0.772*^h^*	–
** 0**	1,308 (82.9%)	154 (85.1%)	5,324 (84.0%)	1,528 (84.8%)	0.489*^h^*	–
**Previous preterm birth (n, %)**	24 (1.5%)	4 (2.2%)	126 (2.0%)	38 (2.1%)	0.564*^g^*	–
**GDM, (n, %)*^bc^***	63 (4.0%)	9 (5.0%)	399 (6.3%)	112 (6.2%)	0.003*^g^*	–
**PIH, (n, %)*^bcde^***	45.7 (2.9%)	3 (1.7%)	285 (4.5%)	79 (4.4%)	0.009*^g^*	–
**Gestational age (week), mean (SD)*^abe^***	39.3 ± 1.5	38.8 ± 1.4	39.0 ± 1.6	39.2 ± 1.6	<0.001*^j^*	0.023
**Birth weight (g), mean (SD)*^b^***	3,405 ± 494	3,441 ± 500	3,443 ± 526	3,406 ± 498	0.009*^f^*	0.976
**Birth weight Z-score, mean (SD)*^b^***	0.41 ± 1.09	0.63 ± 1.16	0.61 ± 1.13	0.46 ± 1.06	<0.001*^f^*	0.415
**Preterm birth (n, %)*^b^***	63 (4.0%)	12 (6.4%)	412 (6.5%)	92 (5.1%)	0.002*^h^*	–
**Low birth weight (n, %)**	50 (3.2%)	5 (2.8%)	215 (3.4%)	52 (2.9%)	0.723*^h^*	–
**Small for gestational age (n, %)**	73 (4.6%)	3 (1.7%)	241 (3.8%)	65 (3.6%)	0.220*^h^*	–
**Large for gestational age (n, %)*^b^***	322 (20.4%)	38 (21.0%)	1,533 (24.2%)	369 (20.5%)	0.001*^h^*	–

NC, naturally conceived; ART, assisted reproductive technology; BMI, body mass index; GDM, gestational diabetes mellitus; PIH, pregnancy-induced hypertension; SGA, small for gestational age; LGA, large for gestational age.

^a^P < 0.05 for the comparison between fertile NC and infertile NC.

^b^P < 0.05 for the comparison between fertile NC and female factor ART.

^c^P < 0.05 for the comparison between fertile NC and male factor ART.

^d^P < 0.05 for the comparison between infertile NC and female factor ART.

^e^P < 0.05 for the comparison between infertile NC and male factor ART.

^f^Welch ANOVA test for difference of all groups and Games–Howell test for differences between groups.

^g^Fisher’s exact test for variance.

^h^Chi-square for variance.

^i^P value for interaction test (between ART treatment and female infertility).

^j^One-way analysis of variance for difference of all groups and Tukey post-hoc test for differences between groups.

### Birth Outcomes

Birth outcomes of the four groups are presented in **Table 1**. Among the four groups, the fertile NC group had the longest GA and the lowest BW (GA: 39.3 ± 1.5 weeks, BW: 3,405 ± 494g, P < 0.05). Compared to the fertile NC group, the infertile NC group had a shorter GA (38.8 ± 1.4 vs. 39.3 ± 1.5 weeks, P < 0.05), but their BW and BW z score showed no significant difference (all P > 0.05). The female factor ART group had a higher BW (3,443 ± 526 vs. 3,405 ± 494 g, P < 0.05) and a higher BW z score (0.61 ± 1.13 vs. 0.41 ± 1.09, P < 0.05) compared to the fertile NC group. A shorter gestational age was observed in the female factor ART group compared to the fertile NC group (39.0 ± 1.6 vs. 39.3 ± 1.5 weeks), which, although statistically significant, did not appear to be clinically significant. However, compared to the infertile NC group, their GA, BW, and BW z score were all comparable (all P > 0.05). Among the four groups, the risks of both LBW and SGA were comparable (all P > 0.05). The female factor ART group showed a higher risk of PTB and LGA compared to the fertile NC group (PTB: 6.5% vs. 4.0%, P < 0.05; LGA: 24.2% vs. 20.4%, P < 0.05). Compared to the infertile NC group, neither the female factor ART group nor the male factor ART group had any significant difference in the four adverse perinatal outcomes (all P > 0.05).

In the pairwise comparison among the fertile NC group, infertile NC group, and female factor infertile group, the differences in birth outcomes only appeared in the comparison between the female factor ART group and the fertile NC group. Since these results suggested an adverse effect of the combination of female infertility and ART treatment, we conducted the interaction test to identify their influence on GA and BW. The interaction between female infertility and ART treatment was significant for GA (P = 0.023) but was not statistically significant for BW (P = 0.976).

When calculating the risk of PTB, LBW, SGA, and LGA, cofounding factors were further adjusted, including female age, female BMI, and pregnancy complications. The adjusted odds ratio (AOR) and 95% confidence interval (CI) of adverse perinatal outcomes are presented in **Table 2**. The increased risk of PTB and LGA still existed in the female factor ART group after adjustment. Since this group can be subdivided according to the cause of infertility, we analyzed the risk of adverse perinatal outcomes within different causes of infertility to investigate the variances of the results. **Table 3** presents the comparison among the fertile NC group, infertile NC group, and subgroups of different infertility causes. When taking the fertile NC group as a reference, there was an increased risk of PTB in the tubal factor ART group, ovulatory dysfunction ART group, and unexplained infertility ART group (AOR and 95% CI: tubal factor ART: 1.49, 1.12–2.00; ovulatory dysfunction ART: 1.87, 1.29–2.72; unexplained infertility ART: 1.88, 1.11–3.17). Besides, our study also showed an increased risk of LGA in the former two groups, with an AOR (95% CI) of 1.28 (1.11, 1.48) in the tubal factor ART group and 1.27 (1.03, 1.57) in the ovulation disorder ART group. However, compared to the infertile NC group, no significant difference in the incidence of any adverse perinatal outcomes was found in any of the four female factor ART groups.

**Table 2:** Risks of adverse perinatal outcomes in infertile groups with or without ART treatment.

Outcomes	Fertile NC	Infertile NC		Female factor ART		Male factor ART	
	aOR (95% CI)	aOR (95% CI)	*P-adj*	aOR (95% CI)	*P-adj*	aOR (95% CI)	*P-adj*
PTB	Ref	1.64 (0.84, 3.21)	0.147	1.56 (1.18, 2.07)	0.002	1.23 (0.88, 1.72)	0.222
	–	Ref	–	0.95 (0.51, 1.77)	0.870	0.75 (0.39, 1.44)	0.384
LBW	Ref	0.85 (0.33, 2.17)	0.728	0.94 (0.68, 1.30)	0.699	0.81 (0.54, 1.21)	0.305
	–	Ref	–	1.10 (0.45, 2.71)	0.838	0.95 (0.37, 2.41)	0.906
SGA	Ref	0.39 (0.12, 1.27)	0.120	0.77 (0.58, 1.03)	0.081	0.72 (0.50, 1.02)	0.063
	–	Ref	–	1.97 (0.62, 6.22)	0.251	1.81 (0.56, 5.86)	0.320
LGA	Ref	1.11 (0.76, 1.64)	0.591	1.27 (1.10, 1.47)	0.001	1.03 (0.87, 1.23)	0.719
	–	Ref	–	1.14 (0.79, 1.65)	0.490	0.92 (0.63, 1.35)	0.676

Models adjusted for female age, female BMI, female PIH, and female GDM.

aOR, adjusted odds ratio; CI, confidence intervals; P-adj, adjusted P‐value; NC, naturally conceived; ART, assisted reproductive technology; PTB, preterm birth; LBW, low birth weight; SGA, small for gestational age; LGA, large for gestational age.

**Table 3:** Risks of adverse perinatal outcomes within different causes of infertility.

Outcomes	Fertile NC		Infertile NC		Tubal factor ART		Ovulatory dysfunction ART		Endometriosis ART		Unexplained infertility ART	
	%	AOR (95% CI)	%	AOR (95% CI)	%	AOR (95% CI)	%	AOR (95% CI)	%	AOR (95% CI)	%	AOR (95% CI)
PTB	4.0	Ref	6.4	1.64 (0.84, 3.20)	6.2*^a^*	1.49 (1.12, 2.00) *^b^*	7.9*^a^*	1.87 (1.29, 2.72) *^b^*	5.7	1.44 (0.56, 3.71)	7.8*^a^*	1.88 (1.11, 3.17) *^b^*
		–		Ref		0.91 (0.49, 1.70)		1.15 (0.59, 2.26)		0.87 (0.29, 2.62)		1.15 (0.53, 2.46)
LBW	3.2	Ref	2.8	0.84 (0.33, 2.15)	3.3	0.91 (0.65, 1.27)	4.2	1.16 (0.73, 1.83)	1.1	0.31 (0.04, 2.29)	3.7	0.99 (0.49, 2.01)
		–		Ref		1.07 (0.43, 2.65)		1.37 (0.52, 3.58)		0.36 (0.04, 3.19)		1.17 (0.39, 3.50)
SGA	4.6	Ref	1.7	0.40 (0.12, 1.28)	4.0	0.82 (0.62, 1.10)	2.7*^a^*	0.53 (0.31, 0.89) *^b^*	5.7	1.21 (0.47, 3.12)	2.2	0.44 (0.19, 1.04)
		–		Ref		2.08 (0.66, 6.58)		1.33 (0.39, 4.57)		3.04 (0.70, 13.13)		1.11 (0.27, 4.52)
LGA	20.9	Ref	22.0	1.11 (0.75, 1.64)	24.3*^a^*	1.28 (1.11, 1.48) *^b^*	25.9*^a^*	1.27 (1.03, 1.57) *^b^*	23.0	1.30 (0.77, 2.20)	21.6	1.06 (0.77, 1.47)
		–		Ref		1.15 (0.79, 1.67)		1.13 (0.76, 1.70)		1.17 (0.63, 2.19)		0.96 (0.60, 1.53)

Models adjusted for maternal age, maternal BMI, maternal PIH, and maternal GDM.

aOR, adjusted odds ratio; CI, confidence intervals; NC, naturally conceived; ART, assisted reproductive technology; PTB, preterm birth; LBW, low birth weight; SGA, small for gestational age; LGA, large for gestational age.

^a^Adjusted P value <0.05 for the comparison with the fertile NC group.

^b^aOR showed significant difference.

## Discussion

According to our data, infertile women with ART treatment had shorter GA and larger BW than the fertile NC women and thus had a higher risk of PTB and LGA. It could not be explained solely by either female infertility or ART treatment but was due to the interaction of both factors. Among different causes of infertility, tubal factors, ovulatory dysfunction, or unexplained infertility increased the risk of PTB, meanwhile the former two factors also increased the risk of LGA.

Several previous studies have reported that ART singletons showed a higher risk of PTB and SGA than NC singletons (6, 22, 23). Our study was partly consistent with the previous findings in PTB and indicated an additional association of ART treatment with LGA. Whether this phenomenon is attributed to maternal infertility or ART treatment has not been determined. Since there would not be any fertile couples seeking ART treatment, the relationship between female infertility and ART treatment was not clarified in the previous studies. In our study, we regarded the male factor ART group as a fertile ART group without female factor infertility. Through the comparison between the male factor ART group and the fertile NC group, we evaluated the influence of ART treatment on the perinatal outcomes in fertile women. The results demonstrated that ART treatment alone showed no association with neonatal weight or PTB in fertile women. This was consistent with Romundstad’s study, which compared the siblings with or without ART treatment and found no difference in perinatal outcomes (24). Similarly, in infertile mothers, ART also did not significantly increase the risk of any poor perinatal outcomes, indicating that maternal infertility was the most likely risk factor. Previous studies did support this point of view (15, 24). However, in our study, this could not be used to fully explain the poor perinatal outcomes in ART-treated infertile women, since the difference in perinatal outcomes in infertile NC women did not reach statistical significance compared with fertile NC women. This means neither infertility nor ART treatment could explain all the increased risks independently. Results of the interaction test in our study suggested that ART treatment might act as a catalyst to exacerbate the impact of infertility on PTB.

According to the present study, unexplained infertility conferred the highest risk of PTB in ART offspring compared with the fertile NC reference, which was consistent with several previous studies (12, 13). Unfortunately, little information about the mechanism had been explored. The possible etiologies may include disturbance in endocrinological balance, genetic defects, immunological deregulation, or potential inflammatory factors (25, 26). Animal and human studies suggested that excess intereluken-6 (IL-6) could suppress reproductive function (27, 28) and lead to unexplained infertility. Elevation of IL-6 in unexplained infertile women may lead to PTB through inflammatory pathways (29–31). However, no association between unexplained infertility and poor perinatal outcomes was found in some other studies (13, 32). The controversy of the results may also be due to the heterogeneous pathophysiological characteristics of unexplained infertility. Thus, the association should be replicated in a more homogeneous unexplained infertile population of a larger sample size.

The ovulatory dysfunction with polycystic ovary syndrome (PCOS) as the predominant cause took the second place of the risks. PCOS was characterized by insulin resistance and hyperandrogenism, showing an increased morbidity of GDM and PIH, which was also confirmed in our research (33–35). Although these complications were suggested as the risk factors of PTB, after adjusting for them, the ovulatory dysfunction group showed an increased risk of PTB. Placenta abnormality was probably the underlying mechanism. Previous studies showed chronic villitis and an increased thickness of stem villi arterial walls in PCOS women. These histological changes could induce PTB through damaged utero-placental circulation (36, 37). However, whether other ovulatory dysfunctions, such as primary ovarian insufficiency (POI), hypogonadotropic hypogonadism (HH), and luteinized unruptured follicle syndrome (LUFS), also conferred the risk of PTB could not be demonstrated in the present study due to the limited sample size.

Additionally, tubal factor infertility could also increase the risk of PTB in ART offspring, although the contribution was slightly lower than that of the above two. A higher risk of PTB in the tubal factor group had been reported in other studies (12, 38), and the result could be explained through the mechanism of inflammation. Most tubal factor infertility was a sequela of pelvic inflammation diseases (PID) (39), and up to 40% of PID cases were due to chlamydia trachomatis infections. During a persistent infection, chlamydial heat shock protein 60 (CHSP60) genes are upregulated and released. Sensitization to the highly conserved region in the HSP of other species could result in the reactivation of lymphocytes (40, 41). This may in turn lead to PTB *via* the proinflammatory response (30, 42).

In our study, LGA was another poor perinatal outcome identified in both ovulatory dysfunction and tubal factor infertility. Consistent with other studies (43, 44), the ovulatory dysfunction was easy to be accepted as a risk factor since PCOS accounted for the majority of patients with ovulation dysfunction. PCOS would directly increase the birth weight of offspring through deteriorating maternal metabolism (45) and indirectly elevate the LGA risk through more frequent usage of frozen embryo transfer (46, 47). Besides, the results of tubal factor infertility were different from those of previous studies which reported a higher risk of SGA (12, 15). The heterogeneity of the studied population was a possible explanation. The result needed to be confirmed in a well-designed large cohort study.

The strength of our study lay in the evaluation of the interaction between ART treatment and maternal infertility. Moreover, the prospective design avoided several biases and provided more solid evidence. Yet, it still had several limitations. First of all, male factor might also have a paternal origin confounding effect on the outcomes and thus might not be an ideal group as the fertile ART group. However, it still provided a reference to some extent since there was no better substitute because of the limitation of ART indication. Secondly, the infertile NC group could only be analyzed as a whole group due to the limited sample size, which restricted the respective analyses of the interaction between ART treatment and each female infertility cause. Thirdly, the relatively small sample size of some etiologies of ovulatory dysfunction, such as POI, HH, and LUFS, limited the analyses of these specific ovulatory dysfunctions and poor perinatal outcomes. In the future, further research is needed in a larger cohort.

In conclusion, our study indicated that ART treatment itself would not deteriorate the perinatal outcomes but could act as a catalyst to amplify the effect of maternal infertility on PTB. Maternal tubal factor, ovulatory dysfunction, and unexplained infertility would confer the risk of PTB after ART treatment, while the former two would also increase the risk of LGA. Clinicians should be alert to the increased risk of adverse perinatal outcomes in certain kinds of infertile couples, provide accurate and credible risk assessments for infertile patients, and provide prevention strategies before or during pregnancy to reduce the occurrence of adverse events.

## Data Availability Statement

The original contributions presented in the study are included in the article/supplementary material, further inquiries can be directed to the corresponding authors.

## Ethics Statement

The studies involving human participants were reviewed and approved by the Reproductive Medicine Ethics Committee, Hospital for Reproductive Medicine Affiliated to Shandong University. The patients/participants provided their written informed consent to participate in this study.

## Author Contributions

YZ performed the statistical analyses and drafted the manuscript. WZ helped with the data analysis. WF and JH provided useful comments to outcome assessment. KH inputted into the revision of the manuscript. LC set up this study and recruited participants. Z-JC contributed to the study concept and design. All authors contributed to the article and approved the submitted version.

## Funding

This study was supported by the National Key Research and Development Program of China (2021YFC2700700), National Natural Science Foundation of China (82171692), Shandong Provincial Key Research and Development Program (2018YFJH0504), Natural Science Foundation of Shandong Province of China (ZR2020MH065), and Taishan Scholars Program for Young Experts of Shandong Province (tsqn201909195). The funders did not have any role in study design, data collection, and data analyses.

## Conflict of Interest

The authors declare that the research was conducted in the absence of any commercial or financial relationships that could be construed as a potential conflict of interest.

## Publisher’s Note

All claims expressed in this article are solely those of the authors and do not necessarily represent those of their affiliated organizations, or those of the publisher, the editors and the reviewers. Any product that may be evaluated in this article, or claim that may be made by its manufacturer, is not guaranteed or endorsed by the publisher.

## Abbreviations

ART, assisted reproductive technology; GA, age of delivery; BW, birth weight; PTB, preterm birth; LBW, low birth weight; LGA, large for gestational age; SGA, small for gestational age; NC, naturally conceived; LMP, last menstrual period; BMI, body mass index; GDM, gestational diabetes mellitus; PIH, pregnancy-induced hypertension; SD, standard deviation; AOR, adjusted odds ratios; CI, confidence interval.
